# Anticytokine therapy for periodontal diseases: Where are we now?

**DOI:** 10.4103/0972-124X.55837

**Published:** 2009

**Authors:** Yogesh Prakash Waykole, S. S. Doiphode, P. S. Rakhewar, Maya Mhaske

**Affiliations:** *Post-graduate Student, Department of Periodontology, C.S.M.S.S Dental College and Hospital, Kanchanwadi, Aurangabad - 431 002, India*; 1 *Professor and Head, Department of Periodontology, C.S.M.S.S Dental College and Hospital, Kanchanwadi, Aurangabad - 431 002, India*; 2 *Associate Professor, Department of Periodontology, C.S.M.S.S Dental College and Hospital, Kanchanwadi, Aurangabad - 431 002, India*

**Keywords:** IL-1, IL-6, proinflammatory cytokines, soluble receptors, TNF

## Abstract

Periodontal destruction is initiated by bacteria that stimulate host responses leading to excess production of cytokines. Anticytokine therapy for periodontal diseases especially targets proinflammatory cytokines, that is, TNF-α, IL-1, and IL-6, because these are essential for the initiation of the inflammatory immune reaction and are produced for prolonged periods in periodontitis. This therapy aims to bind the cytokines with the receptors present on target cells such as the fibroblasts. The three basic treatment strategies are: (1) neutralization of cytokines, (2) blockage of cytokine receptors, and (3) activation of anti-inflammatory pathways, such as, immune-suppressive pathways.

This new therapy can act as a host response modulator in the control of inflammatory diseases of gums and may provide the basis for new molecular therapeutic approaches to the treatment of periodontitis.

## INTRODUCTION

**Figure 1:**
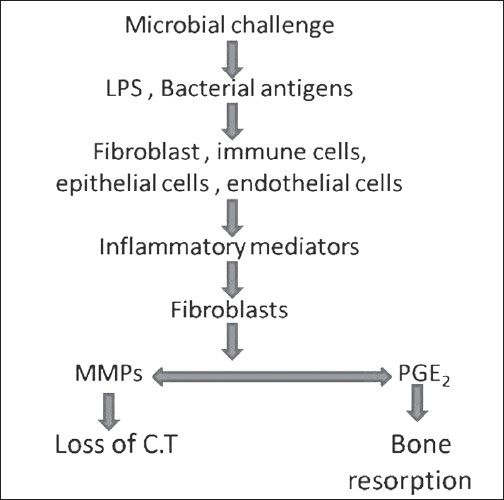
Model for pathogenesis of periodontitis

Periodontitis is an inflammatory disease fundamentally initiated by chronic bacterial infection.[1,2] Current data suggest that a small group of predominantly gram-negative anaerobic or microaerophilic bacteria within the biofilm are often associated with disease initiation and progression. The microbial challenge consisting of antigens, lipopolysaccharides, and other virulence factors stimulates host responses. Host reactions to these infecting agents result in the release of inflammatory mediators including proinflammatory cytokines (IL-1, IL-6, TNF-α) and prostaglandins (PGE_2_), which can promote extracellular matrix destruction in the gingiva and stimulate bone resorption[3] [Figure 1].

Although these immune and inflammatory host reactions are essential for host defense against bacterial inflammation, excessive and prolonged reaction is harmful for the functional periodontal tissue. Regulation of the immune and inflammatory reaction, in addition to controlling infection in the periodontal tissue, may be one of the methods to prevent and treat periodontal diseases.

Traditional periodontal treatment includes debridement of infectious matter and inflamed tissue. However, some situations where traditional therapy alone is not successful include:[4]

- Patients with non-microbial risk factors, which are difficult to reduce or eliminate, such as, smoking, diabetes, and so on
- Factors that are beyond the clinician's ability to control, such as, genetic predisposition
- A specific group of periodontal disease susceptible individuals

Therefore, in these instances, the use of the anti-cytokine therapy, in conjunction with anti-biofilm treatments, may prove to be advantageous.

## CYTOKINES

Cytokines are a category of signaling proteins and glycoproteins, and are used extensively in cellular communication.

## NOMENCLATURE

### Early names

- Monokines and lymphokines (to describe source)
- Now they are called just Cytokines.

### Interleukin

A cytokine that is produced and acts on leukocytes. (now used generically)

### Chemokine

Cytokine that induces chemotaxis. (Chemotactic cytokines)

## CLASSIFICATION

Cytokines are classified as[5]

**Table:** 

FAMILY	MEMBERS
Chemotactic	IL-8, MIP-1, MCP-1, RANTES
Pro-inflammatory	IL-1α, IL-1β, TNF-α, IL-6
Anti-inflammatory	IL-1Ra, IL-4, IL-10
Growth factor	PDGF, EGF, FGF, IGF, VEGF
immunoregulatory	IFN-γ, IL-2, 4, 5, 7

MIP - macrophage inflammatory protein, MCP - monocyte chemotactic protein, RANTES - regulated upon activation, normal T cell expressed and secreted, IL-1Ra - interleukin 1 receptor antagonist, PDGF - platelet derived growth factor, EGF - epidermal growth factor, FGF - fibroblast growth factor, IGF - insulin-like growth factor, VEGF - vascular endothelial growth factor, IFN - interferon)

Anticytokine therapy for periodontal diseases mainly targets TNF-α, IL-1β, and IL-6, because they are essential for the initiation of inflammatory immune reactions and are produced for prolonged periods in inflammatory disease.

## MECHANISM OF ACTION

**Figure 2:**
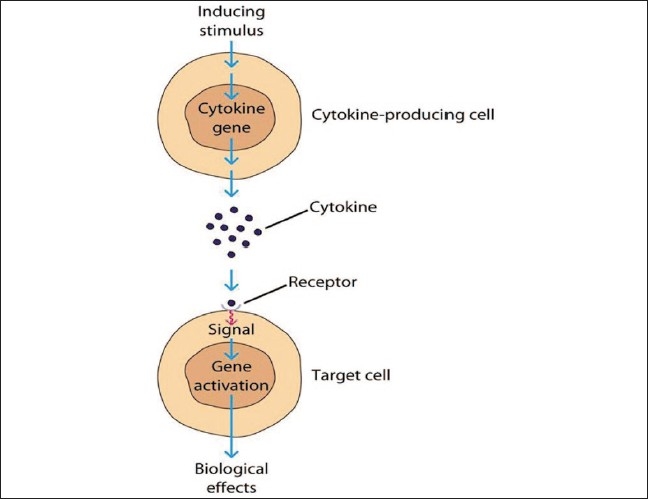
Mechanism of action of cytokines

The microbial challenge consisting of antigens, lipopolysaccharides, and other virulence factors, acts as an inducing stimulus. They stimulate cytokine producing cells. Upon stimulation, activation of the cytokine gene occurs and it releases cytokine in the solution. This cytokine binds the receptors present on the target cell. Upon binding, gene activation occurs in the target cell, which releases secondary mediators (i.e., MMPs and PGE_2_). These mediators are responsible for loss of connective tissue and bone resorption [Figure 2].

## MECHANISM FOR CYTOKINE-RECEPTOR INTERACTION

**Figure 3:**
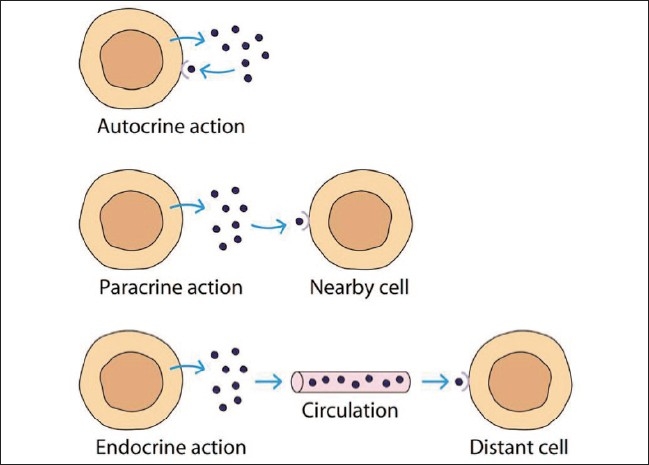
Mechanism for cytokine-receptor interaction

Cytokines may react with the receptor present on cell through different mechanisms [Figure 3]

1. Autocrine — cytokine binds to the receptor on the cell of origin
2. Paracrine — cytokine binds to the receptor present on the adjacent cells
3. Endocrine — cytokine is released into circulation and can act at distant sites
4. Juxtacrine — membrane-bound cytokine that acts on receptors on either the same cell or adjacent cells

## CYTOKINE RECEPTORS

The effects of cytokines are mediated by membrane-bound receptors, which are present on the surface of target cells.

**Table:** 

Cytokines	Memberne-bound receptors
IL-1β	IL-1 RI
	IL-1RII
	(IL-1RAcP)
TNF-α	TNF-RI
	TNF-RII
IL-6	IL-6R
	(gp130)

Periodontal therapy for periodontal disease aimed at inhibiting the binding of cytokines to receptors is present on target cells such as fibroblasts.

## DOWNREGULATION OF CYTOKINES

Downregulation of cytokines is mainly brought about by three mechanisms:

### i. Cytokine receptor antagonists

Cytokine receptor antagonists bind to the receptor present on the target cell and prevent the cytokine from binding to the target cell. Therefore, there is no activation of the target cell.

Example: IL-1 receptor antagonist. (IL-1Ra) Production of IL-1Ra appears to play a role in regulating the intensity of inflammatory responses.

### ii. Soluble cytokine receptors

Soluble cytokine receptors are derived from the proteolytic cleavage of the extracellular domain of cell-bound cytokine receptors. Soluble receptors can be found in blood and extracellular fluid.

The downregulation of cytokine is brought about by mainly two mechanisms

1. Downregulation — Soluble receptors bind to the cytokine in solution and prevent signaling.
2. Transactivation — Soluble receptors bind the cytokine and docks on otherwise non-responsive cells and activate them.

**Table:** 

Cytokines	Soluble receptors
IL-1β	sIL-1R
TNF-α	sTNF-RI
	sTNF-RII
IL-6	sIL-6R

Out of all these soluble receptors only sIL-6R is an agonist in function, the rest are all antagonist in function and bring about the downregulation of cytokines.

#### Anti-cytokine antibodies

Anti-cytokine antibodies are also antagonist in function and they lower down the levels of cytokines.

**Table:** 

Cytokines	Antibodies
TNF-α	Anti-TNF Ab
IL-6	Anti-IL-6 Ab

## IMPLICATION FOR PERIODONTAL DISEASES

Rheumatoid arthritis is one of the best disease models suitable for anti-cytokine therapy. The principles of this strategy have been reviewed previously.[6] There are three basic therapeutic strategies:

- Neutralization of cytokines
- Blockage of cytokine receptors
- Activation of anti-inflammatory pathways such as immunosuppressive pathways

## COMMERCIALLY AVAILABLE PREPARATIONS

### Infliximab (Remicade)

TNF-α is a special target molecule known for its neutralizing properties, therapeutics. Anti-TNF-α antibodies has effectively attenuated or prevented inflammation of arthritis in experiment models.[7]

Infliximab is a chimeric IgG monoclonal antibody. The term “chimeric” refers to the use of both mouse (murine) and human components of the drug. The drug also has been successfully used in:

- Ankylosing spondylitis: 5 mg per kg
- Crohn's disease: 5 mg per kg
- Psoriatic arthritis: 5 mg per kg
- Rheumatoid arthritis: 3 mg per kg
- Psoriasis: 5 mg per kg

### Etanercept (Enbrel)

TNF-α can also be neutralized with genetically engineered sTNF-α-RII.[8] Etanercept (enbrel) is a fusion protein. It links human soluble TNF receptor to the Fc component of human IgG1. It has been successfully used in some autoimmune diseases:

1. Ankylosing spondylitis
2. Juvenile rheumatoid arthritis
3. Psoriasis
4. Psoriatic arthritis
5. Rheumatoid arthritis

### Anakinra (Kineret)

It is an interleukin-1 (IL-1) receptor antagonist. It competitively inhibits the binding of IL-1 to the Interleukin-1 type receptor.[9] Anakinra blocks the biological activity of naturally occurring IL-1, including inflammation and cartilage degradation.

It is used for the management of signs and symptoms of rheumatoid arthritis.

Several studies have been carried out for periodontal diseases and have shown the potential of using IL-1β and TNF-α antagonists, to reduce tissue destruction in periodontal diseases.[10–13] These researchers applied exogenous sIL-1RI and sTNF-RII to the gingival tissues of non-human primates with experimental periodontitis and found inhibition of inflammatory cell infiltration, alveolar bone loss, and loss of tissue attachment.

## PHARMACOLOGICAL AGENTS STILL UNDER RESEARCH

There are certain pharmacological agents with potential host modulation action, but more studies are yet required toward their therapeutic use in treatment of periodontal diseases.

### 1. Recombinant human interleukin-11 (rhIL-11)

Interleukin 11 has been shown to have anti-inflammatory effects by inhibition of TNF-α and other proinflammatory cytokines.[14] IL-11 directly minimizes tissue injury through the stimulation of a tissue inhibitor of metalloproteinases-1 (TIMP-1).[15]

Based on these previous studies, Martuscelli *et al.* carried out a study using recombinant human interleukin-11 (rhIL-11) in the treatment of ligature-induced periodontitis, in dogs.[16] They found a significant reduction in the rate of clinical attachment loss and radiographic bone loss after an eight-week period of rhIL-11 administration, twice a week.

### 2. Disruption of cell signaling pathways

Strategies for preventing cell activation seek to inhibit the intracellular transduction of signals produced when ligands bind to their membrane receptors. Signal transduction pathways are mainly activated by cytokines, but also by other factors, such as, bacterial proteins, lipoproteins or environmental stress. Mitogen-activated protein kinase (MAPK) pathway is one of the signal transduction pathways closely involved in inflammation. MAPKs are divided into three families — the extracellular signal-regulated kinases (ERK1/2), c-jun N-terminal kinases (JNKs), and p38.

In recent years, the identification of proinflammatory signal transduction pathways has suggested new therapeutic targets. As these are shared by several cytokines, their inhibition will probably prove more powerful than the current treatment strategies.

#### i. Cytokine suppressive anti-inflammatory drugs (CSAIDS) / p38 inhibitors

The role for p38 MAPK, in various stages of inflammation, has prompted the production of several imidazole compounds capable of inhibiting p38 (RWJ 67657, VX- 745, and others). These inhibitors are called CSAIDs and are responsible for the *in vitro* and *in vivo* inhibition of LPS-induced TNF-α expression.[17]

In the experimental arthritis models, p38 inhibitors prevent the development of arthritis and bone erosions. Parasrampuria DA *et al*. tested RWJ 67657 in human volunteers.[18] After a single dose of RWJ 67657, the serum levels of the proinflammatory cytokines TNF-α, IL-6, and IL-8 were decreased by 90% compared with their plasma peak.

Kirkwood *et al*. showed that p38α selective mitogen activated the protein kinase inhibitor, which prevents periodontal bone loss in rats.[19]

#### ii. JNK inhibitors

The specific JNK inhibitor, SP600125, not only diminishes the production of TNF-α, interferon-γ, IL-6, COX-2, and matrix metalloproteinases, but also decreases the joint destruction in the adjuvant arthritis model.[20]

To date, no human trials have been initiated with these inhibitors. With JNK, it seems that both isoforms (JNK1 and JNK2) must be inhibited to produce an anti-inflammatory effect.

### Resolvins

They are compounds that are made by the human body from the omega-3 fatty acids, eicosapentaenoic acid (EPA) and docosahexaenoic acid (DHA). Compounds derived from EPA are designated as Resolvins of the E series (RvE1), and those biosynthesized from DHA are denoted as Resolvins of the D series (RvD).

Resolvins stimulate the resolution of inflammation through multiple mechanisms, including preventing neutrophil penetration, phagocytosing apoptotic neutrophils to clear the lesion, and enhancing clearance of inflammation within the lesion to promote tissue regeneration.[21,22,23]

Hasturk *et al*.[24] showed that, in a rabbit model of human periodontal disease, RvE1 prevents the initiation and progression of tissue destruction.

These results support the hypothesis that both EPA- and DHA-derived resolvins have therapeutic potential in resolving periodontal inflammation and restoring the tissues' health.

## DRAWBACKS

1. Periodontitis is an inflammatory disease fundamentally initiated by chronic infection. When inflammation is inhibited, the immune system is also downregulated. This increases the risk of microbial infection.
2. Opportunistic infection has been reported when TNF-α was neutralized for rheumatoid arthritis therapy.[25]
3. The screening of latent infectious diseases, such as tuberculosis, should be performed before using this type of anti-cytokine therapeutic.
4. With antimicrobials, caution must be taken to prevent inapparent infection, without inflammatory symptoms, when anti-cytokine therapy is performed. If anti-cytokine therapy is applied to periodontal treatment, we may use chemical plaque control reagents such as chlorhexidine gluconate in addition to mechanical control.

## CONCLUSION

In this era of molecular biology where research has been focused on the genetic level of analysis, treatment should be focused on eliminating the root cause.

Periodontal advancement should be diverted toward the use of anti-cytokine therapy in the near future.
